# Does It Matter If Students (Dis)like School? Associations Between School Liking, Teacher and School Connectedness, and Exclusionary Discipline

**DOI:** 10.3389/fpsyg.2022.825036

**Published:** 2022-03-03

**Authors:** Linda J. Graham, Jenna Gillett-Swan, Callula Killingly, Penny Van Bergen

**Affiliations:** ^1^Centre for Inclusive Education, Queensland University of Technology (QUT), Brisbane, QLD, Australia; ^2^School of Education, Macquarie University, Sydney, NSW, Australia

**Keywords:** disadvantage, teacher-student relationships, school connectedness, dis/engagement, suspension and exclusion

## Abstract

School liking is an important factor in student engagement, well-being, and academic achievement, but it is also potentially influenced by factors external to the individual, such as school culture, teacher support, and approaches to discipline. The present study employed a survey methodology to investigate the associations between school liking and disliking, teacher and school connectedness, and experiences of exclusionary discipline from the perspective of students themselves. Participants included 1,002 students (Grades 7–10) from three secondary schools serving disadvantaged communities. Results indicated clear differences between students who like and dislike school in terms of their preferred school activities and school disciplinary history, with students who disliked school experiencing overall lower school connectedness. Moreover, students who disliked school experienced less positive relationships with their teachers, and this was even more pronounced for students who had been previously suspended. The findings reveal key differences between students who do and do not like school, differences that may be masked by typical research approaches. This research indicates the need for more nuanced, student-informed approaches to inclusive school reform.

## Introduction

“School liking” is a term used to describe when students’ perceptions of, and feelings about school are positive—at least, most of the time. Liking for school has been found to play an important role in children’s adjustment to school (Ladd and Burgess, 2001; Walker and Graham, 2019), and is related to both student engagement and scholastic achievement more broadly (Ladd et al., 2000; Riglin et al., 2013). Although most children and young people will dislike some aspects of school, at some point in time, this type of dislike tends to be transient and constrained to certain subjects, issues, or individuals. Dislike for school, however, can become pervasive, such that some students who dislike school, dislike it completely. This *pervasive* form of dislike can result from academic difficulties, which can induce a self-perception of academic failure/incompetence (Murray and Mitchell, 2013; Graham et al., 2016), and is associated with classroom disruption (King et al., 2006; Graham et al., 2016; Walker and Graham, 2019) and truancy (Attwood and Croll, 2015). Disliking school is strongly related to the students’ perceptions of their school’s psychological climate (Frazier et al., 2015), with prior research detecting a positive association between secondary school students’ dislike for school and experiences of peer harassment, including regular teasing, name calling, and exclusion (Eisenberg et al., 2003). There are potentially other factors related to school climate and culture that affect school liking; however, the literature on school liking is limited.

Students’ (dis)like for school is often incorporated into broader measures of student adjustment or attitudes. For instance, one relatively well-known measure of school adjustment is the *School Liking and Avoidance Questionnaire* (SLAQ; Ladd and Price, 1987), which is considered to provide an overall measure of how well students are adjusting to school. The SLAQ, however, is principally used with students in the primary (or elementary) phase of schooling and is not appropriate for use with adolescents. For this group of young people, school liking may be measured effectively by asking students whether they like school, and their history of liking or disliking a school (e.g., Graham et al., 2016). Previous research using this approach for adolescents with and without a history of disruptive behavior has found reliable differences between students who do and do not like school with the use of just one item: “Do you like school?” (Graham et al., 2016). The most common reasons students provided for disliking school in Graham et al.’s (2016) study were “schoolwork” and “teachers,” with most students who dislike school reporting that they most commonly get in trouble with teachers for “not following instructions” and “not doing work” (Graham et al., 2016). These students were also significantly less likely to remember any teachers with whom they had a positive relationship (Van Bergen et al., 2020), and had experienced many difficulties with learning, as well as multiple long suspensions of up to 20 days per suspension, eventually resulting in exclusion/expulsion from school (Graham and Buckley, 2014). Such evidence suggests that students who dislike school may be more likely to experience conflict and have poorer quality relationships with teachers, feel less connected to school, and be subject to higher rates of exclusionary discipline, which is precisely the opposite of what these students need to stay in and succeed at school (McGrath and Van Bergen, 2015).

### Teacher–Student Relationships, School Connectedness, and Exclusionary Discipline

Given the frequency of students’ interactions with teachers, it is perhaps not surprising that close and supportive student–teacher relationships predict students’ liking for a school (Roorda et al., 2011). When a student feels personally respected and cared for by their teacher, they tend to like school more (Hallinan, 2008). They also become more engaged in learning and experience greater academic gains (Hughes and Kwok, 2007; Roorda et al., 2017). In contrast, teacher–student relationships high in conflict are associated with lower levels of school-liking (Ladd and Burgess, 2001). He et al. (2019), for example, found a relationship between teachers’ use of emotional punishments, higher depression, and lower school connectedness. Teacher–student relationship quality also strongly predicts students’ connectedness to school, which is itself linked to positive student outcomes and acts as a buffer against other risks (Monahan et al., 2010).

School connectedness encompasses a student’s sense of school belonging, their acceptance in the social environment of their school, and the degree to which they feel personally respected and supported (Goodenow, 1993). It therefore includes, but extends, beyond the teacher–student relationship, with other contextual and systemic factors also implicated (e.g., school policies related to inclusion and discipline, peer acceptance and support, and pedagogical practices that support students’ autonomous decision-making, learning, and success). Being connected to school is positively associated with educational achievement (Niehaus et al., 2012; Pate et al., 2017) and school progression, including the likelihood of completing secondary school (Bond et al., 2007). It is also positively associated with emotional wellbeing and negatively associated with mental health problems, such as depression (Shochet et al., 2006; Bond et al., 2007). Longitudinal research shows that higher school connectedness buffers against poor mental health (Foster et al., 2017), later conduct problems (Loukas et al., 2010), risk-taking behaviors (Resnick et al., 1997; Chapman et al., 2011), and adverse health behaviors, such as cigarette smoking (Bonny et al., 2000).

Research shows that school suspension and exclusion can have a negative impact upon students’ sense of social belonging and on their trust in school authority figures (Pyne, 2019; Jacobsen, 2020). For example, drawing on the National Longitudinal Study of Adolescent Health with more than 75,000 students, McNeely et al. (2002) found that school connectedness was reduced in schools with poor classroom management and those which “temporarily expel” students for minor infractions, in comparison to schools with positive classroom management and more tolerant disciplinary approaches. Findings that relate exclusionary school discipline to lower school connectedness are not simply a reflection of challenging student behavior. While the comorbid effects of behavior and discipline can be difficult to disentangle, emerging research highlights the positive impact of programs that focus on improving the school climate and reducing the use of exclusionary discipline. Huang and Cornell’s (2018) comprehensive study of 310 middle schools in Virginia showed that schools that were authoritative, rather than authoritarian, and which had strict *but fair* discipline and a focus on positive student–teacher relationships, had fewer out of school suspensions, and a stronger sense from students that their teachers care about them.

Importantly, school connectedness and positive teacher–student relationships are not dependent on funding or whole-of-system reform, but can instead be enhanced through inclusive school reform at both local and regional levels. It is rare, however, that student–teacher relationships or students’ connectedness to and liking for school is the focus of reform, despite their strong association with student engagement, learning, and behavior. This is surprising given the implications for inclusive school reform decisions. *If* what is working for some students in terms of school culture and climate is not working for *all*, it makes sense to pay attention to *who* is not liking and connecting to school, *why*, and whether improving key elements of school culture and climate, such as teacher–student relationships and school connectedness, may help improve students’ liking for school, decrease conflict with teachers, and reduce exclusionary discipline.

The aim of this study was to better understand factors driving classroom disruption, disengagement from school, and exclusionary discipline in complex secondary schools serving disadvantaged communities. In this manuscript, we investigate associations between school liking, teacher–student relationship quality, connectedness to school, and students’ experience of detention, suspension, and exclusion. Central to the initiation of the project was the aim of reducing teacher–student conflict due to an altercation between a student and a teacher in one of the participating schools, which resulted in a student being permanently excluded and a principal seeking more just solutions. The project was expanded to include two additional high-need secondary schools serving disadvantaged communities with the support of the respective region and funding from the Queensland Government. The research focused on Grades 7–10 (junior secondary school) as these grades have been found to record the highest number of suspensions, exclusions, and enrollment cancelations (Graham, 2018). Consistent with the philosophy of inclusive education, student voice was paramount to the project ethos. The final design reflected this with a large-scale survey aimed at gauging differences in experiences and perspectives between students across cohorts, followed by individual interviews using purposeful sampling to represent students with a history of behavioral incidents. This manuscript reports on the findings from the student survey which was administered to 1,002 students in Grades 7–10 across the three participating schools.

## Materials and Methods

### Participants

All students in Grades 7–10 for whom parent consent had been confirmed were invited to participate in an electronic survey by the respective school principal or their delegate (e.g., Head of Grade or Project Liaison). Responses were received from 1,002 students in Grades 7–10. Two of the schools had an Index of Community Socio-Educational Advantage (ICSEA) 1 standard deviation below the national mean of 1,000, and the third school was on the mean (Table 1).

**TABLE 1:** School demographics and distribution of student participants.

School ID	Enrollments Year 7–12	LBOTE (%)	Indigenous (%)	ICSEA range (2017)	% cohort in lowest SEA quartile (%)	Student survey Grades 7–10 (*n* = 1,002)
School A	700+	2	11	900–949	58	273
School B	1,500+	12	16	900–949	52	531
School C	500+	23	8	1,000–1,049	23	198

*LBOTE, language background other than English.*

*All schools in Australia are given an ICSEA score: a calculation of the relative affluence of the school community (Australian Curriculum, Assessment and Reporting Authority [ACARA], 2020). ICSEA has a mean of 1,000 and a standard deviation of 100. Note, as geographic information or single ICSEA scores could reveal the identity of the schools, only ICSEA ranges have been provided here. Socio-economic advantage (SEA) scores represent the socio-economic distribution of students in the school.*

In Queensland, students in Grade 7 are aged between 12 and 13 years; Grade 8 are 13–14 years; Grade 9 are 14–15 years; and Grade 10 are 15–16 years. Table 2 contains demographic details about the participants in the sample.

**TABLE 2:** Descriptive characteristics of students grade level, age, and gender.

		*n*	%	M*_*age*_* (SD)
Grade level	7	314	31.3	12.6 (1.1)
	8	226	22.6	13.5 (0.9)
	9	260	25.9	14.5 (0.8)
	10	202	20.2	15.2 (1.1)
Gender	Male	463	46.2	
	Female	503	50.2	
	Other	36	3.6	

There were no significant differences in student characteristics between the three schools: the gender distribution (male, female, or other) did not vary, χ^2^(4) = 8.48, *p* = 0.075, nor did the number of students who liked or disliked school, χ^2^(2) = 3.89, *p* = 0.143. Neither detentions [χ^2^(2) = 0.26, *p* = 0.880] nor expulsions [χ^2^(2) = 0.46, *p* = 0.796] differed among the three schools, but prior suspensions did, χ^2^(2) = 21.31, *p* < 0.001, Cramer’s *V* = 0.15, as follow-up *z* tests (Bonferroni-adjusted) indicated that one of the schools had a significantly lower proportion of students who had received suspensions (*p* < 0.05).

### Materials and Procedure

The research was conducted according to the ethical standards of the institutional and national research committees. The study was approved by the Queensland University of Technology (QUT) Human Research Ethics Committee, and approval to conduct the research was obtained from the Queensland Department of Education.

Participants completed the survey between April 1 and September 25, 2017 within class time and under the supervision of their classroom teacher. The survey included demographic questions about age, gender, and grade. Using yes/no response options, students were asked whether they liked school, whether they had always liked school (for those who responded yes, they liked school), when their dislike began (for those who responded no, they did not like school), and whether they had ever received a detention or been suspended or excluded. To indicate what they liked most and least about the school, students were also asked to select their most liked aspect of the school from a list of seven options arranged in alphabetical order: Breaktime, Friends, Learning, Homework, Music/Art/Drama, Sport, and Teachers. They were then asked to select their least liked aspect of school from a list of six options: Schoolwork, Teachers, Uniform, Peers, Homework, and Discipline policy (e.g., school rules). These questions were drawn from a previous study investigating severely disruptive school behavior with 96 students aged 8–17 years in New South Wales, Australia (Graham et al., 2016).

Finally, the survey instrument also included two validated scales that have been used in the research literature to measure students’ connectedness to their teachers and to school. The first of these is the 6-item School Support Scale (Hanson and Kim, 2007), which was adapted from the National Longitudinal Study of Adolescent to Adult Health Study (Hanson and Kim, 2007) and taps caring adult relationships by “directly ask[ing] students about caring adults at their school” (Furlong et al., 2011, p. 995) with statements like “My teacher really cares for me” (Table 3). The responses were made on a 5-point Likert scale ranging from never (1) to always (5), and high scores indicate higher connectedness to teachers. The scale has good reliability (Cronbach’s α = 0.83). One additional item, “My teacher has time for me,” was added to the school support scale items based on common responses to an interview question in previous research probing student’s perspectives on what makes positive teacher–student relationships (Graham et al., 2016). This item was significantly correlated with all other items on the scale (*r* = 0.37–0.64).

**TABLE 3:** Connectedness to teacher and connection to school scale items.

*School support scale* (Hanson and Kim, 2007)	*Connection to school scale* (Brown, 1999)
My teacher…	*Belief/Power subscale* (1) Adults at this school listen to students’ concerns
(1) Really cares about me	(2) Adults at this school act on students’ concerns
(2) Tells me when I do a good job	(3) I have many opportunities to make decisions at my school
(3) Notices when I’m not there	(4) The principal at this school asks students about their ideas
(4) Always wants me to do my best	(5) I am comfortable talking to teachers at this school about problems
(5) Listens to me when I have something to say	(6) The rules at my school are fair
(6) Believes that I will be a success	(7) We do not waste time in my classes
(7) Has time for me*	(8) Students of all racial and ethnic groups are respected at my school
	(9) When students have an emergency, someone is there to help
	(10) It pays to follow the rules at my school
	*Commitment subscale*
	(11) I can be a success at this school
	(12) My schoolwork helps in things that I do outside of school
	(13) I can reach my goals through this school
	*Belonging subscale*
	(14) I can be myself at this school
	(15) I feel like I belong at this school
	(16) I have friends at this school

*This item was added to the scale by the researchers.

The second scale used is the 16-item Connection to School Scale (Brown, 1999), which measures how connected a student feels to their school and includes items such as “Adults at this school LISTEN to students’ concerns” (Table 3). Responses are given on a 4-point Likert scale ranging from strongly disagree (1) to strongly agree (4). The overall scale has good reliability (Cronbach’s α = 0.86). It has three subscales – belief/power^1^ (α = 0.81; 10 items), commitment (α = 0.85; three items), and belonging (α = 0.51; three items). High scores on this measure indicate high levels of school connection.

### Analytical Strategy

No items on the survey had more than 1% missing values. Quantitative analyses were conducted for participants with complete data on all relevant variables, excluding two participants who provided incongruous responses to some questions (*n* = 993). We first conducted analyses that assess the association between liking school vs. disliking and (i) grade level, (ii) gender, (iii) most- and least-enjoyed aspects of school, and (iv) exclusionary discipline (suspensions, exclusions, or detentions). Given the categorical nature of these variables, we employed Chi-square tests of independence for analysis and used Cramer’s *V* to indicate effect size. For the analysis of most-liked aspects of school, a Fisher–Freeman–Halton exact test was applied, as the expected cell counts were below 5. Significant Chi-square tests with more than two categories per variable were further investigated with *z* tests for comparing two proportions (applying a Bonferroni correction for multiple comparisons).

We next conducted analyses that compared students’ perceptions of school support and school connectedness according to school liking and their experience of exclusionary discipline. As the dependent variables were continuous in nature (means scores on the school support and school connectedness scales and subscales), we employed both mixed design and between-groups univariate analyses of variance (ANOVA), adopting a multivariate approach for the analysis of repeated measures variables. Cohen’s *d* and partial eta square ($\eta_{p}^{2}$) were used to indicate effect size. Where follow-up comparisons have been conducted, a Bonferroni correction was applied. Means and bivariate correlations for all scales are provided in Appendix Table 1.

## Results

### School Liking

Although most students indicated that they liked school (66.5%), approximately one-third (33.5%) did not. School-liking varied by grade level, χ^2^(3) = 9.10, *p* = 0.028, Cramer’s *V* = 0.10. Follow-up z tests showed that Grade 7 (72.8%) had a significantly higher percentage of school-likers than Grade 9 (61.8%), indicating a decline in school liking over time. Grade 8 (66.1%) and Grade 10 (63.1%) did not differ significantly from other groups. School-liking was significantly associated with gender, χ^2^(2) = 18.28, *p* < 0.001, Cramer’s *V* = 0.14, such that a lower proportion of students identifying as “Other” (35.3%) indicated that they liked school, compared to those who were male (64.9%) or female (70.1%; *p* < 0.05).

#### History of School-Liking

Of the students who liked school, approximately half (46.1%) reported that they had always liked school. Students who disliked school were asked at what point they started disliking school, where the options provided were “Kindy–Grade 2,” “Grades 3–6,” “Grades 7 and 8,” or “Grades 9 and 10.” For students in Grades 8, 9, and 10, the highest percentage of students indicated Grades 7 and 8 as the starting point, followed by Grades 3–6, and Kindergarten–Grade 2 (Table 4). Grade 7 students predominantly indicated Grade 3–6 as the point where they began to dislike school. We note that Grade 8 was the first year of high school in Queensland until 2015 when Grade 7 transferred to the secondary phase of schooling (Graham, 2018). Therefore, Grade 9 and 10 students who nominated Grade 8 as the point at which they began disliking school had also commenced high school that year.

**TABLE 4:** Grade level when students (%) began to dislike school, split according to current grade level.

	Grade level when school dislike began			
Current grade	K - Grade 2	Grade 3–6	Grade 7 and 8	Grade 9 and 10
Grade 7 (*n* = 85)	35.3	47.1	17.6	
Grade 8 (*n* = 76)	21.1	27.6	51.3	
Grade 9 (*n* = 99)	14.1	21.2	53.5	11.1
Grade 10 (*n* = 73)	23.3	26.0	35.6	15.1

#### Most- and Least-Liked Aspects of School

**FIGURE 1:**
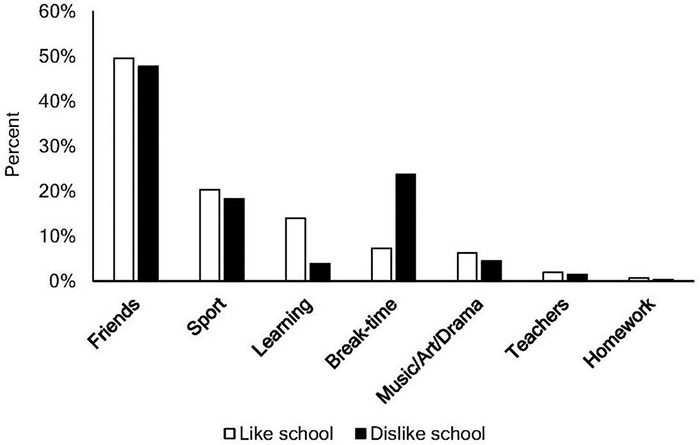
Most liked school activities for school-likers (white columns) and school-dislikers (black columns).

Students’ responses regarding their most- and least-liked aspects of school are displayed separately for school likers and school dislikers, respectively. For students’ most-liked aspect of school, “Friends” was the most selected category and “Homework” the least selected category for both groups (Figure 1). Nonetheless, significant differences in students’ most-liked aspects of school emerged, Fisher–Freeman–Halton test, *p* < 0.001. To determine the nature of these differences, we conducted follow-up *z* tests with a Bonferroni adjustment. “Learning” was selected as the most-liked element by a significantly higher percentage of school likers than dislikers (*p* < 0.05), whereas “Break-time” was preferred by a significantly higher percentage of school dislikers than likers (*p* < 0.05). No other categories significantly differed based on school liking.

**FIGURE 2:**
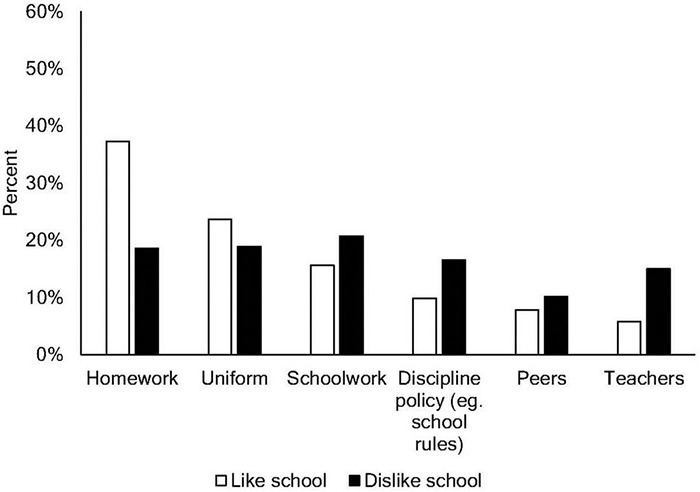
Least liked school activities for school-likers (white columns) and school dislikers (black columns).

There were also distinct patterns of response to the “least-liked” elements of school (Figure 2) based on overall school-liking, χ^2^(5) = 61.34, *p* < 0.001, Cramer’s *V* = 0.25. Follow-up *z* tests indicated that a higher percentage of school dislikers than likers selected “Schoolwork,” “Teachers,” and “Discipline Policy” (*p*s < 0.05) as their least-liked aspect of school. School-likers most frequently selected “Homework” as their least-liked school element, and this proportion significantly exceeded the proportion of school dislikers selecting the same category (*p* < 0.05).

#### Differences in Connection to Teachers and School Liking

**FIGURE 3:**
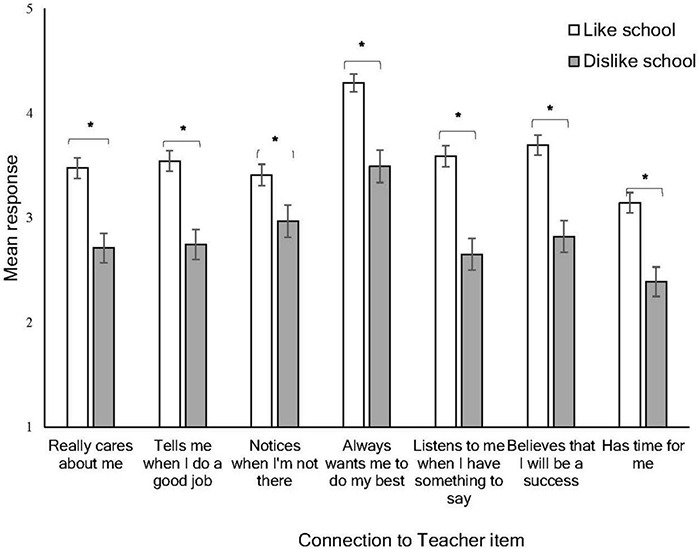
Mean responses to the school support scale items according to whether students like or dislike school. Error bars indicate 95% CIs. *Statistical significance.

Students who liked school had a mean response of 3.67 (SD = 0.93) on the 6-item student support scale, and those who did not like school had a mean of 2.9 (SD = 1.04). Individual items on the scale were of particular theoretical interest when considering differences among school likers and dislikers. Therefore, students’ responses on each scale item are displayed in Figure 3, according to whether they like or dislike school. A 2 × 7 mixed ANOVA was conducted to investigate differences in each of the items among school likers and dislikers. There were significant main effects of school liking, *F*(1, 991) = 140.45, *p* < 0.001, $\eta_{\text{p}}^{2}$ = 0.124, and the student support scale items, Wilks’ λ = 0.57, *F*(6, 986) = 122.25, *p* < 0.001, $\eta_{\text{p}}^{2}$ = 0.427. There was also a significant interaction, Wilks’ λ = 0.98, *F*(6, 986) = 4.15, *p* < 0.001, $\eta_{\text{p}}^{2}$ = 0.025, indicating that responses to the scale items varied as a function of whether or not students liked school.

To follow up this interaction, Bonferroni-adjusted *post hoc* comparisons were conducted, which revealed that students who dislike school rated each item significantly lower than those who like school (*p*s < 0.001). The significant interaction may therefore be explained by the fact that the degree of difference between school-likers and dislikers in their rating varied based on each item, as depicted visually in Figure 3. The magnitude of the difference between groups was smallest for the item “Notices when I’m not there” (*d* = 0.32), which represents a small effect (Cohen, 1988). All other items had medium effect sizes (*d* = 0.58–0.69), with the largest observed for the item “Listens to me” (*d* = 0.69). The highest-rated item for both groups was “Always wants me to do my best,” while the lowest, again for both groups, was “Has time for me.” It is notable that on every item except “Always wants me to do my best,” the average ratings of students who dislike school fell below the midpoint of the response scale (3).

#### School Liking and School Disciplinary Experiences

Not liking school was associated with detentions, χ^2^(1) = 56.28, *p* < 0.001, Cramer’s *V* = 0.24. Of the students who reported not liking school, 71.2% had received a detention in the past 12 months. For students who did like school, only 46.1% had received a detention. Not liking school was also associated with suspension, χ^2^(1) = 91.45, *p* < 0.001, Cramer’s *V* = 0.30, with 41.4% of students who disliked school having previously received a suspension, compared to only 14.2% of school likers. School liking was also related to previous expulsions, χ^2^(1) = 18.72, *p* < 0.001, Cramer’s *V* = 0.14, with 8.4% of school dislikers reporting that they had previously been expelled, compared to only 2.4% of school likers.

#### Does Connection to Teachers Vary Based on School Discipline History and School Liking?

**FIGURE 4:**
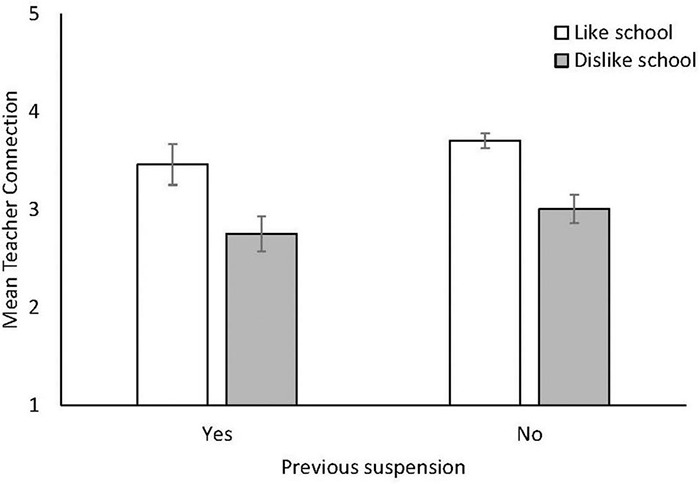
Mean score on the school support scale according to whether students like or dislike school, and whether they have received a suspension. Error bars indicate 95% CIs.

A 2 × 2 between-groups ANOVA was conducted to examine whether teacher connectedness, as measured by the school support scale (using the 6-item mean score^2^), varied as a function of either school liking or suspension history (Figure 4). There were significant main effects of both suspension, *F*(1, 989) = 10.02, *p* = 0.001, $\eta_{\text{p}}^{2}$ = 0.011 and school liking, *F*(1, 989) = 85.85, *p* < 0.001, $\eta_{\text{p}}^{2}$ = 0.080, but no significant interaction, *F*(1, 989) < 1, *p* = 0.958, $\eta_{\text{p}}^{2}$ = 0.000. Both a dislike of school and a history of suspensions are linked to reduced connectedness to teachers, and these negative effects are additive, such as students experience lower connection to their teachers when they dislike school, and even more so when they have experienced suspension.

#### Connection to School and School Liking

**FIGURE 5:**
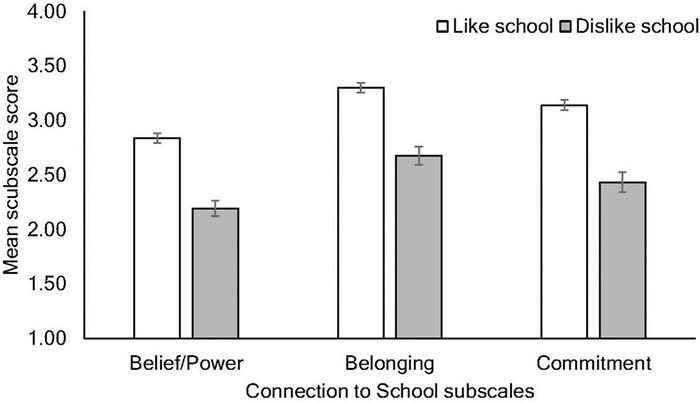
Mean responses to the connection to school subscales according to whether students like or dislike school. Error bars indicate 95% CIs.

Figure 5 displays mean scores on the three connection to school subscales (belief/power, belonging, and commitment) according to school liking. To determine whether these components of school connection varied according to school liking, a mixed 2 × 3 ANOVA was conducted, with school liking as the between-subjects factor and connection to school subscales as the within-groups factor. There was a significant main effect of school liking, *F*(1, 991) = 320.06, *p* < 0.001, $\eta_{\text{p}}^{2}$ = 0.244, indicating that school-likers rated all subscales significantly higher than dislikers. There was also a significant effect of school connection subscale, Wilks’ λ = 0.64, *F*(2, 990) = 280.01, *p* < 0.001, $\eta_{\text{p}}^{2}$ = 0.361, and no interaction, Wilks’ λ = 0.97, *F*(2, 990) = 1.89, *p* = 0.151, $\eta_{\text{p}}^{2}$ = 0.004. Follow-up pairwise comparisons of the school connection’s main effect revealed significant differences between each subscale (*p*s < 0.001) where scores were highest on the belonging subscale, followed by the commitment subscale, and then the belief/power subscale.

Although items related to belief and power are loaded onto one factor in the analysis reported by Brown et al. (2000), they represent conceptually distinct and potentially meaningful constructs that are of a particular interest when considering differences among students who like or dislike school. Hence, a supplementary analysis was performed to see whether school liking influenced responses to items that were theorized to align with “Power” and “Belief.” Separate subscale means were calculated using the four items aligning to each theorized factor (see Brown et al., 2000), and a 2 × 2 mixed ANOVA was conducted, with subscale (power, belief) as the within-subjects factor and school liking as the between-subjects variable. There were significant main effects of both subscale, Wilks’ λ = 0.88, *F*(1, 991) = 133.45, *p* < 0.001, $\eta_{\text{p}}^{2}$ = 0.119 and school liking, *F*(1, 991) = 223.38, *p* < 0.001, $\eta_{\text{p}}^{2}$ = 0.184, indicating that scores on the power subscale were significantly lower than on the belief subscale, and that school dislikers had significantly lower scores than likers on both. There was also a significant interaction, Wilks’ λ = 0.98, *F*(1, 991) = 22.21, *p* < 0.001, $\eta_{\text{p}}^{2}$ = 0.022.

**FIGURE 6:**
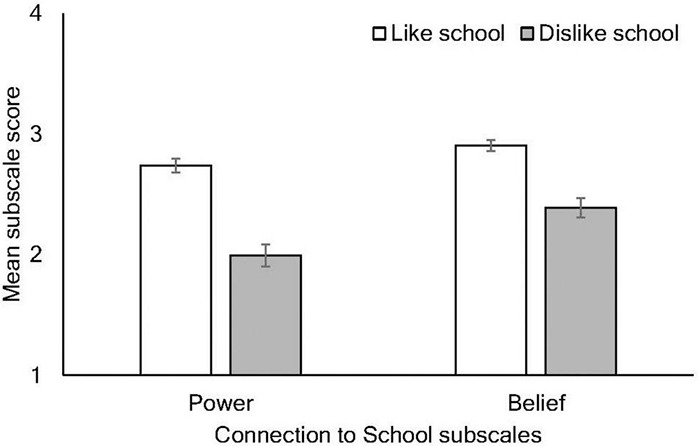
Mean responses to the connection to school subscales (power and belief) according to whether students like or dislike school. Error bars indicate 95% CIs.

To follow up the significant interaction, separate univariate ANOVAs were conducted to investigate group differences for the power and belief scales separately. There were significant differences between school likers and dislikers on both subscales, with the larger effect observed for the power subscale [power: *F*(1, 991) = 198.08, *p* < 0.001, $\eta_{\text{p}}^{2}$ = 0.167; belief: *F* (1, 991) = 136.23, *p* < 0.001, $\eta_{\text{p}}^{2}$ = 0.121]. Hence, school likers and dislikers diverged to a greater extent on items related to power compared to those related to belief (see Figure 6).

## Discussion

The present study examined associations between school liking, teacher–student relationships, school connectedness, and experience of exclusionary school discipline through a survey of 1,002 junior secondary school students from Grades 7–10 to better understand factors driving classroom disruption and disengagement from school. Findings revealed stark differences between students who like and dislike school. While some of these differences might be expected, in that, students who dislike school may be more likely to engage in conflict with teachers and might, therefore, experience higher rates of exclusionary discipline, our findings present a more nuanced account.

Approximately two-thirds of the students reported liking school overall, and one-third reported disliking school. While the percentage of school likers decreased between Grades 7 and 9, dislike is also reported by most students as a relatively recent phenomenon. Older students most commonly reported beginning to dislike school in Grade 7 and 8, raising questions about the transition to secondary school, whereas younger Grade 7 students most commonly reported onset in the upper primary years. Taken together with evidence that only half the school likers group have always liked school, school liking and disliking appear variable across the school years, with negative attitudes more prominent during or after periods of transition.

The shift from primary to secondary school is a time of significant adjustment for young people, both interpersonally and academically (Evans et al., 2018), representing a pivotal developmental window during which students require greater support (Lester et al., 2013). Secure student–teacher relationships have been shown to be protective against the potential psychological and academic impacts of these transitions (Longobardi et al., 2016, 2019). This potential was recognized in Queensland when Grade 7 transitioned from the primary to the secondary phase of schooling in 2015, and a junior secondary model with core subject teachers was implemented to ensure Grade 7s had fewer teachers to navigate (Pendergast et al., 2015). While the decline in school liking from a high point in Grade 7 through Grades 8–10, with the lowest point in Grade 9, may provide some evidence of the relative success of this approach, it is important to note that more than one-quarter (27.2%) of Grade 7s still did not like school. Our findings suggest that school disliking increases over time and, like out-of-school suspensions and exclusions (Graham, 2018) may peak in Grade 9.

To better understand the factors that drive school (dis)liking, we mapped students’ most-liked and least-liked aspects of school. Students who like and dislike school were consistent in their response that “friends” were one of the most-liked aspects of school, with approximately half of each group choosing this option. For students who did not select friends as their most-liked aspect, however, differences between school likers and dislikers emerged: more school likers reported “learning” as their most-liked aspect of school, whereas more school dislikers reported “break-time.” This finding is perhaps not surprising as break-time offers an escape from regular school activities. Consistent with previous research (Graham, 2016), schoolwork and teachers are also mentioned frequently by school dislikers who also report disliking discipline policy significantly more than school likers.

School dislikers were more likely than school likers to have received detention in the past 12 months and were also more likely to have previously received a suspension or expulsion from school. This finding is consistent with prior research which indicates an association between negative perceptions of school and experience with exclusionary discipline (Huang and Anyon, 2020). A growing body of evidence attests the harmful impacts of out-of-school suspension on student outcomes, negatively affecting relationships with peers (Jacobsen, 2020) and teachers (Quin, 2019), and academic achievement (Noltemeyer et al., 2015; Hwang, 2018). Moreover, suspension is known to exacerbate rather than remediate the behaviors for which it is issued (Quin and Hemphill, 2014; Amemiya et al., 2020; Wiley et al., 2020). It is worth noting that not all students who disliked school in the present study had prior experiences of school suspension or exclusion. Likewise, while school likers were less likely than school dislikers to have experienced these disciplinary practices, there remained nevertheless a substantial proportion of students who liked school but also had a history of detention or suspension. Other non-disciplinary factors are therefore also likely to be relevant to school liking but, in combination, it appears that relationship building and school belonging have substantive impacts on students’ enjoyment of school.

We therefore also examined students’ perceptions of their connectedness to teachers and school, and how these related to their overall school-liking. School dislikers provided significantly lower ratings to every aspect of teacher–student relationships in comparison to likers, although notably, there were some items on which both likers and dislikers appeared to provide lower responses, namely, their perception of the teacher having time for them. This important link between perceived teacher support and adolescents’ attitudes toward school has been identified in a recent meta-analysis, where teacher support was identified as one of the strongest predictors of a student’s sense of belonging at school (Allen et al., 2018). Connectedness to teachers overall was lowest for those students who had previously been suspended and who disliked school. While the directionality of this relationship cannot be inferred in a cross-sectional design, existing research highlights the detrimental effect that suspension can have on student–teacher relationships (Quin and Hemphill, 2014), with school disciplinary practices serving to erode student trust in school authority figures (Pyne, 2019). Positive teacher–student relationships, in contrast, have been associated with reduced risk of suspension and school disengagement (Quin, 2017), and schools which have sought to improve student–teacher relationships through targeted intervention have seen suspension rates halved (Okonofua et al., 2016). Lastly, higher ratings on some individual items should not necessarily be interpreted as positive. For example, the second highest-rated item by school dislikers was that their teacher “Notices when I’m not there;” however, this may not be desirable from the perspective of a student who dislikes school and who actively avoids attending.

In terms of school connectedness, school dislikers provided significantly lower ratings than likers overall; all students, regardless of whether they liked school, provided the highest ratings for school belonging, followed by commitment, and finally the belief/power subscale. In previous research using this scale, Brown et al. (2000) found that each subscale was negatively correlated with substance use, with the strongest association evidenced for belief/power. All subscales were positively correlated with school participation, with the strongest association shown for the belonging subscale. Finally, school grades significantly correlated with the commitment subscale. These different components of school connectedness therefore provide an important insight into students’ school-related experiences and outcomes. Importantly, the belief/power subscale includes items that tap students’ perception of their power to influence adult decision-making, a key factor in the development of a school’s culture and climate (Cohen et al., 2009).

The present study included a supplementary analysis of this subscale, separating items related to belief and power, indicated that students provided lower responses on the power subscale, and the disparity between likers and dislikers was even more pronounced. It is fascinating that the students in this study who reported disliking school, and who may engage in behavior that disrupts the school environment potentially resulting in conflict with teachers, appear to perceive themselves as having less power than school likers do, or perhaps cannot see the relative influence of their actions on their school’s climate. Importantly, our analyses found no significant differences on any measure between the three schools, suggesting that the identified differences between school likers and dislikers are stable across schools. These differences may therefore reflect systemic, as opposed to idiosyncratic, issues affecting school liking and, with it, students’ connectedness to both school and teachers.

Our findings suggest that inclusive school reform should include specific measures to improve school cultures and specifically enable teachers with more time to connect with their students. We note that this time needs to be well spent on building rapport and trust, as well as providing more individualized support to students who experience difficulties with schoolwork, rather than simply “checking in” with or monitoring students’ whereabouts. Interventions targeting relationship-building have been found to have a positive impact upon school liking, as well as academic achievement (Miller et al., 2017); however, it is important that these types of initiatives are implemented universally to promote positive teacher–student relationships more broadly, with specific strategies to engage more intensively with specific groups. While many schools have implemented universal policies and practices to promote student engagement and wellbeing, our findings suggest that these may not address the specific concerns of individuals or distinct groups. Our findings show that the concerns of students who report a dislike for school differ from those of students who do like school but, also importantly, their specific concerns could be masked by school satisfaction and engagement surveys that aggregate results for all students. This could potentially drive lower perceptions of power, which we suggest are reflected by responses to the two power items given the lowest ratings by school dislikers: “the principal at this school asks students about their ideas” and “adults at this school act on students’ concerns.”

Finally, findings from this research also suggest that systemic reform to reduce schools’ reliance on exclusionary discipline may help to support any gains from interventions aimed at improving school liking, teacher–student relationships and school connectedness. For policy and practice ideas, Australian schools and systems need look no further than the United States, where there has been considerable reform activity over the last decade. Strong limits have been placed on out-of-school suspension to prevent both overuse and inappropriate use due to decades of empirical evidence attesting to its harmful effects, particularly in young children for whom suspension is banned in a growing number of districts (Graham et al., 2020). Importantly, discipline reform in many public-school systems, such as Chicago Public Schools, has been coupled with systemic inclusive school reform (Graham et al., 2022). Approximately one quarter of the largest school districts in the United States have now implemented schoolwide frameworks to deliver evidence-based prevention and intervention practices and programs, and have recorded improvements in school safety, and student connectedness and academic achievement as a result.

The limitations of the present study are that it employed survey methodology in only three secondary schools in Queensland serving students from disadvantaged communities at one time point, which may have played a part in the similarity in findings across schools. Future research involving a much larger sample of schools would allow for multilevel modeling and more fine-grained comparisons between schools to determine whether the similarities detected in this study hold between demographically and geographically distinct schools. This type of analysis is important in policy terms, as it may be that there are differences in the relational experiences of students attending disadvantaged vs. advantaged schools or metropolitan vs. regional/remote schools. Further, a cross-sectional study lacks capability to determine the direction of the associations between school liking, exclusionary discipline, and teacher/school connectedness. Future research is needed not only to disentangle this relationship but also to test whether inclusive school reform, including interventions to improve the quality of relationships between teachers and students who do not like school, has a positive impact on these students’ connectedness to school, attitudes toward teachers, attendance, and classroom behavior.

## Conclusion

This study examined associations between school liking, teacher and school connectedness, and student experiences of school discipline. Understanding more about school culture from student perspectives may improve outcomes for students and teachers, particularly for students who do not like school and who are already at risk of disengagement and/or early school leaving. This is an important pursuit given the association between school connectedness, educational achievement, and emotional wellbeing evidenced in prior studies (e.g., Niehaus et al., 2012; Pate et al., 2017). The current study found student perspectives on their school experiences are connected to the quality of their relationships with teachers, as well as school disciplinary practices. Importantly, this study found that students experience lower connection to their teachers when they dislike school, and even more so when they have experienced suspension. This finding adds to a considerable body of literature emphasizing connections among exclusionary discipline, attitudes toward school, and student–teacher relationships (Hallinan, 2008; Allen et al., 2018; Huang and Cornell, 2018; Huang and Anyon, 2020). Results from this study further highlight the role that student–teacher conflict may have in influencing these outcomes, suggesting that intervention strategies targeting student–teacher relationships may be the most beneficial in ameliorating outcomes for both students and teachers (e.g., Okonofua et al., 2016).

The study also contributes to existing research through providing a deeper understanding of when and how students may shift from transient to pervasive dislike for school, and in particular how these changing perspectives of school coincide with key school transitions. While origins and commencement of school dis/liking differ, the transition into high school appears prominent for the participants in the current study. This finding, paired with significant differences in teacher and school connectedness for school dis/likers, points to the importance of cultivating positive relationships to support student enjoyment of school, school belonging, and student transition experiences. Understanding when students may begin to dislike school and some of the conditions surrounding this change in perception of school offers possible points for targeted teacher support strategies to mitigate these effects, with the possibility to change student trajectories and experiences at the school level as a result. For example, implementing unified, evidence-based delivery of school-wide supports through a Multi-Tiered Systems of Support (MTSS) framework may better support positive student experiences and connection with school from the outset.

Revisiting the question posed in the title of the manuscript of whether it matters if students (dis)like school, the results of the current study suggest that yes, it does matter. The nuanced account of student experiences presented through the findings in the current study offer a deeper understanding of the school experience from the perspectives of students themselves, particularly those at risk of disengaging from school, and emphasizes the need for more attention to be directed toward reform efforts focusing on student connectedness to teachers and school. Importantly, these are aspects of the school experience that can be controlled at the school level and should not require additional funding, although release from face-to-face teaching to allow teachers time to connect with and provide additional support to students at risk of disengaging would be a wise investment. Future research could test whether inclusive school reform with specific strategies to enhance student–teacher relationship quality helps to improve students’ liking for, connectedness to, and behavior at school.

## Data Availability Statement

The original contributions presented in the study are included in the article/supplementary material, further inquiries can be directed to the corresponding author.

## Ethics Statement

The studies involving human participants were reviewed and approved by Queensland University of Technology Human Research Ethics Committee and the Queensland Department of Education. Written informed consent to participate in this study was provided by the participants’ legal guardian/next of kin.

## Author Contributions

LG and JG-S conceptualized and managed the project, collected, cleaned and coded the data. CK conducted all quantitative analyses with advice from PV. All authors contributed to wrote the manuscript and approved the submitted version.

## Author Disclaimer

The views expressed herein are those of the authors and are not necessarily those of the Queensland Department of Education.

## Conflict of Interest

The authors declare that the research was conducted in the absence of any commercial or financial relationships that could be construed as a potential conflict of interest.

## Publisher’s Note

All claims expressed in this article are solely those of the authors and do not necessarily represent those of their affiliated organizations, or those of the publisher, the editors and the reviewers. Any product that may be evaluated in this article, or claim that may be made by its manufacturer, is not guaranteed or endorsed by the publisher.

## Footnotes

1 Originally, Brown (1999) constructed items to represent four theorized factors (Belief, Power, Commitment, and Belonging). However, subsequent factor analysis (Brown et al., 2000) indicated that items relating to Belief and Power loaded onto the same factor, as did one of the original Belonging items (item 5). We therefore collapse these items into one subscale in the present study.

2 The same pattern of significant results was observed in a secondary analysis which used as its dependent variable the 7-item mean (including the additional Teacher Connection item in the calculation of the subscale).

## Appendix

**APPENDIX TABLE 1:** Means and bivariate correlations among student support and school connection scales.

	Mean (SD)	Bivariate correlations			
		(1)	(2)	(3)	(4)
(1) Student Support Scale (6 item mean)	3.4 (1.0)	1	0.539**	0.394**	0.515**
(2) School connection: belief	2.6 (0.7)		1	0.600**	0.712**
(3) School connection: belonging	3.1 (0.7)			1	0.574**
(4) School connection: commitment	2.9 (0.8)				1

****Correlation is significant at the 0.01 level (2-tailed).*
